# Introducing a Novel Combined Acetabuloplasty and Chondroplasty Technique for the Treatment of Developmental Dysplasia of the Hip

**DOI:** 10.7759/cureus.21787

**Published:** 2022-01-31

**Authors:** Hamed Yazdanshenas, Firooz Madadi, Mohsen Sadeghi-Naini, Firoozeh Madadi, Amador Bugarin, Mohammad Sadegh Sabagh, Caroline Hing, Arya Nick Shamie, Frances J Hornicek, Eleby Rudolph Washington III

**Affiliations:** 1 Orthopedic Surgery and Family Medicine, University of California Los Angeles David Geffen School of Medicine, Los Angeles, USA; 2 Surgery, Orthopedic Surgery, and Family Medicine, Charles R. Drew University of Medicine and Science, Los Angeles, USA; 3 Orthopedic Surgery, Shahid Beheshti University of Medical Sciences, Akhtar Hospital, Tehran, IRN; 4 Neurosurgery, Shahid Beheshti University of Medical Sciences, Imam Hossein Hospital, Tehran, IRN; 5 Anesthesiology, Shahid Beheshti University of Medical Sciences, Tehran, IRN; 6 Orthopedic Surgery, Charles R. Drew University of Medicine and Science, Los Angeles, USA; 7 Orthopedic Surgery, University of California Los Angeles, Los Angeles, USA; 8 General, Visceral and Transplantation Surgery, University of Heidelberg, Heidelberg, DEU; 9 Trauma and Orthopedics, St George's University Hospitals NHS Foundation Trust, London, GBR; 10 Orthopedic Surgery, University of California Los Angeles David Geffen School of Medicine, Los Angeles, USA

**Keywords:** hip, chondroplasty, early open reduction, acetabular coverage, developmental dysplasia of the hip

## Abstract

Background: The aim of the treatment of developmental dysplasia of the hip (DDH) is to maintain a concentric reduction. We describe a novel approach to treat DDH that involves improvement of cartilaginous acetabular coverage, involves the preservation of the secondary ossification center of the acetabulum, and is adjunctive to early open reduction.

Methodology: Thirty-nine children (40 hips) aged six to 18 months were included in the study. Open reduction with chondroplasty was performed during the same surgery. Patients were followed up for 15 years with both clinical and radiological assessments. At the final follow-up, all patients were graded as good or excellent according to Severin’s classification.

Results: The mean age at reduction was 11.9 months (range: 8-16). The mean preoperative acetabular index (AI) was 43.43 (range: 40-48). After the operation, mean AI decreased to 16.97 (P < 0.0001, 95% confidence interval (CI) = 16.24-17.70). AI improved significantly during growth (mean AI changes 13.50, P < 0.0001, 95% CI = 12.65-14.34). The mean lateral center-edge (CE) angle at skeletal maturity was 32.94° (SD = 4.16°). Mild avascular necrosis (AVN) was observed in two hips with involvement of the epiphysis and was of Kalamchi grade 1.

Conclusion: Chondroplasty in conjunction with open reduction can yield a concentric reduction with improved acetabular coverage that facilitates acetabular remodeling that is sustained until skeletal maturity. Prompt correction through this procedure may help to improve the development of the hip and lead to near normal function as demonstrated by improved mean AI and Severin scores at the last follow-up. With low complication and reoperation rates, this procedure could be considered as a surgical treatment option for DDH in patients between the age of six and 18 months.

## Introduction

Developmental dysplasia of the hip (DDH) consists of subluxation or dislocation of the growing hip joint. The main aim of the treatment of DDH is to maintain a concentric reduction until skeletal maturity. If a concentric, stable reduction is maintained, the acetabulum has the potential to remodel to the normal configuration until maturity [1,2].

Most DDH patients are currently detected via screening before four months of age and treated with a Pavlik harness or abduction splint, depending upon age. If concentric stable reduction cannot be achieved by this method in two to three months, then the treatment has failed [3]. In contrast, patients who are diagnosed late (after 18 months of age) or who develop DDH during growth are often treated with an open reduction of the hip and a Salter’s innominate osteotomy [4].

The earliest age at which an open reduction can safely be carried out is controversial [5]. Early surgical reduction implies that fewer adaptive changes to the femoral head, acetabulum, and capsular structure have taken place, which reduces the time required for these structures to return to their normal configuration. Alternatively, if surgical reduction is performed before 18 months, the cartilaginous shallow acetabulum may cause instability of the relocated hip, which necessitates another pelvic operation to achieve a stable and concentric reduction of the hip [6].

In the present study, we introduce the operative technique, chondroplasty, as a novel adjunct to early open reduction in children aged between six and 18 months with hip dysplasia. With the addition of chondroplasty, cartilaginous acetabular coverage may improve, allowing for a more robust concentric reduction with less morbidity when compared to traditional osteotomy.

## Materials and methods

Patient characteristics

Thirty-nine consecutive children aged between six and 18 months were included in this study for six years. Twenty-six of them had no previous treatment, and 13 had failed initial treatments, which include seven patients with multiple diaper attempts, four with Pavlik harness, and two with casting. Patients were not screened with regard to the presence or absence of an ossific nucleus. Dislocated hips secondary to neuromuscular causes were excluded. The eligible patients were operated on in the same time period and followed until skeletal maturity (range: 13-17 years) with assessments occurring preoperatively, postoperatively, and at the time of skeletal maturity. Written informed consent was obtained prior to inclusion in the study. The study protocol was approved by Akhtar Hospital Institutional Review Board (IRB) in Tehran (Approval number: 31-1992).

Operative technique

All procedures were carried out and supervised by one orthopedic surgeon. Open reduction was performed through an anterolateral approach with an extension of the proximal limb of the incision up to the iliac crest. A split was made between the tensor fascia lata and the gluteus medius, exposing the anterior capsule. A T-shaped capsulotomy was performed, and the folded labrum was everted and preserved. At the level of the lesser trochanter, iliopsoas tenotomy was performed. The hypertrophied ligamentum teres was detached from the femoral head. From the acetabulum, the transverse ligament was incised, the pulvinar was removed, and any other obstruction to reduction was removed as well. The femoral head was then reduced and maintained by capsulorrhaphy. Following the open reduction of the femoral head, derotational osteotomy was also performed if anteversion exceeded 45°.

Chondroplasty

**Figure 1:**
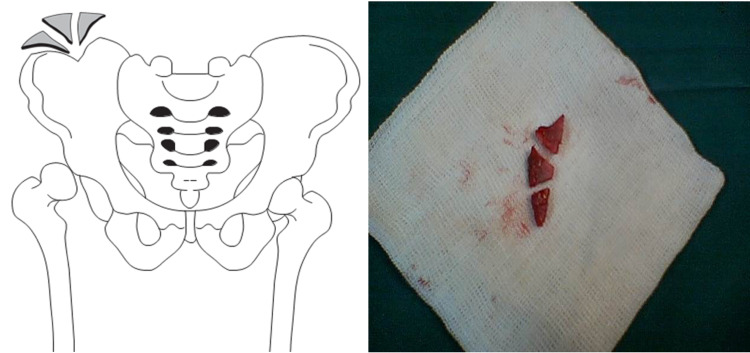
Autograft preparation A triangular segment of bone was harvested from the iliac crest and divided into three triangular pieces.

**Figure 2:**
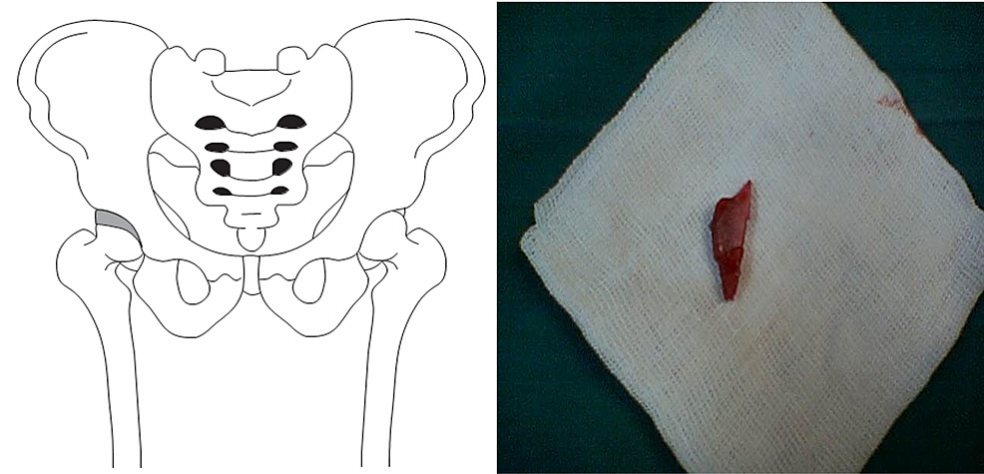
Acetabular roof reconstruction by chondroplasty After the reduction of the femoral head, the artificial space of chondrolabral space was stabilized by suturing the capsular rim and was then filled with the prepared wedge bone graft.

**Figure 3:**
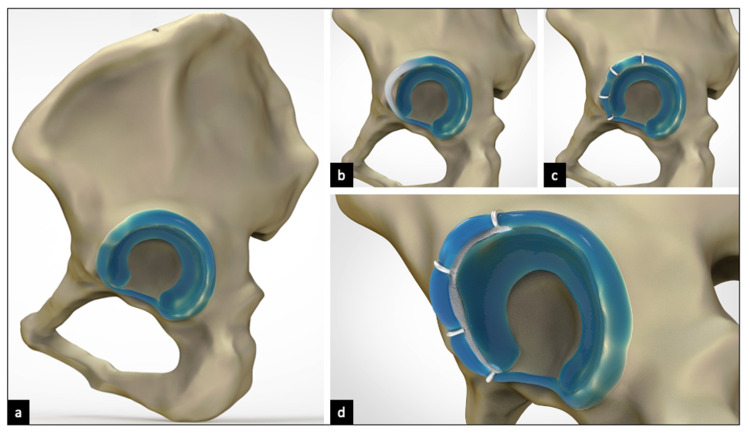
Three-dimensional models illustrating chondroplasty technique (a) Pelvis with acetabular soft tissues after iliac crest bone harvest. (b) After reduction of the femoral head, the anterolateral chondrolabral tissue (white portion) was identified, gently separated, and stretched anteriorly and laterally. (c) The artificial space was stabilized by suturing the capsular rim and chondral labral tissue at its displaced position. (d) Space was then filled with the prepared wedge bone graft to achieve additional chondrolabral tissue stretch.

A triangular segment of bone was harvested from the iliac crest and divided into three triangular pieces (Figure 1). Two centimeters of an anterior crown of the iliac crest were preserved to keep the normal shape of the pelvis. After reduction of the femoral head, the anterolateral cartilaginous acetabular rim was identified. Then the chondrolabral tissue was gently separated from the acetabulum at the capsular insertion on the lateral acetabular ring epiphysis. The separation followed the contour of the acetabulum. The tissue was “teased” and stretched anteriorly and laterally. After the expansion of the cartilaginous rim tissue, a depth of 8-10 mm and a width of 5-7 mm were acquired. This artificial space was then stabilized by suturing the capsular rim with 2-5 nonabsorbable Ticron sutures (depending on the size of the graft and area). The space was then filled with a prepared wedge bone graft that was placed along the anterolateral acetabular rim where the chondrolabral tissue was teased out using small joker dental freer elevator instruments. The graft allowed the chondral tissue to be displaced more anteriorly and laterally and to sustain the tension of the expanded cartilaginous acetabular rim. Although the volume of the space was increased in the older patient due to the increase in the dimension of the hip (larger structures), the percentage increase in the chondrolabral area volume was greater in the younger patient (Figures 2, 3).

Postoperative treatment

A hip spica cast was applied for six weeks after surgery. Thereafter, an abduction broomstick cast was applied for another six weeks. The patients were examined with clinical and radiologic assessments every three months in the ﬁrst year, every six months for the next two years, and annually thereafter. Despite multiple scheduled follow-up visits, only assessments in pre-operation, post-operation, and at skeletal maturity were summarized and reported in this study.

Radiologic assessment

Anteroposterior (AP) and frog-leg radiographs were taken in standard supine position preoperatively, postoperatively, and at each follow-up visit thereafter. The final radiological assessment at skeletal maturity consisted of a weight-bearing AP radiograph of the pelvis. The radiographic analysis consisted of an assessment of concentric reduction and acetabular dysplasia based on an acetabular index (AI), angle of Tönnis (defined as the angle formed between the roof or iliac portion of the acetabulum and a horizontal line passing through the tri-radiate cartilage) [7], Severin’s classification (defined by the radiographic appearance and center-edge angle of the affected joint [Table 1]) [8], and the lateral center-edge (CE) angle of Wiberg (defined as the angle between a vertical line and a line from the center of the femoral head to the most lateral part of the acetabulum) [9]. Grading as Severin I or II was classiﬁed as “negative” for residual dysplasia. Severin III to VI hips were classiﬁed as “positive” for residual dysplasia and considered failures of treatment.

**Table 1:** Severin’s classification in patients older than 14 years of age

Order	Radiographic appearance	Center-edge angle	
1	Normal	1a	>25°
		1b	20° to 25°
2	Moderate deformity of femoral head, femoral neck, or acetabulum	2a	>25°
		2b	20° to 25°
3	Dysplasia without subluxation	-	<20°
4	Moderate subluxation	4a	≥0°
	Severe subluxation	4b	<0°
5	Femoral head articulates with pseudoacetabulum in the superior part of the original acetabulum	-	-
6	Redislocation	-	-

Patients were assessed for numerous complications such as avascular necrosis (AVN), apparent leg length discrepancy, impaired range of motion after skeletal maturity, and gait abnormalities. AVN was graded according to the Kalamchi’s classification system [10] with grade I reﬂecting changes to the epiphysis, grade II indicating the involvement of the lateral part of the growth plate, grade III indicating central physeal damage, and grade IV showing an injury to the entire proximal femoral physis and epiphysis.

Apparent leg length was defined as the distance from the anterior superior iliac spine to the medial malleolus. The range of motion of the hip was assessed and compared mainly for flexion and abduction. Gait was classified as normal, mild limping, and severe gait dysfunction.

Statistical analysis

The primary outcome was defined as the AI. The statistical analysis was performed using the IBM SPSS Statistics v20.0 (IBM Corp., Chicago, IL, USA). A paired sample T-test was used to test for any statistically significant diﬀerences between any of the three follow-up assessments. The level of significance was set at 0.05. Severin classification, lateral CE angle of Wiberg, hip range of motion, and complications were also recorded.

## Results

Thirty-five out of 39 patients completed the follow-up period. Four patients (five hips) were lost to follow-up and were excluded from the analysis. The mean follow-up period was 15 years (range: 13 to 17), and the mean age of the patients at the most recent follow-up was 16 years (range: 14 to 18). The mean age at reduction was 11.9 months (range: 8 to 16).

Radiologic and clinical analysis

The initial mean AI was 43.43 (range: 40 to 48), indicating pronounced acetabular dysplasia. After the operation, there was a marked improvement of the dysplasia with a mean decrease in AI of 16.71 (P < 0.0001, confidence interval [CI] 95% = 15.92-17.5). Patients were followed until skeletal maturity and showed significant AI improvement during growth with mean AI changes of 13.71 (P < 0.0001, CI 95% = 12.78-14.64). Radiologic results at skeletal maturity categorized acetabular dysplasia as Severin class 1a in 24 hips (68%), 1b in two hips (5.7%), 2a in six hips (17%), and 2b in three hips (8.5%). The mean lateral CE angle at skeletal maturity was 32.94° (SD: 4.16°). Range of motion was assessed in two dimensions whereby mean flexion and abduction were 122.8° (SD 4.8°) and 43.9° (SD: 4.2°), respectively. In skeletal maturity, six patients (17%) had limping on examination.

Complications

No serious early complications occurred after any of the 40 open reductions with chondroplasty procedures. AVN occurred in two hips with involvement of the epiphysis; the two hips were classified as Severin 2a with Kalmachi grade 1 AVN. Three patients needed a derotational osteotomy during open reduction; two of them were classified as Severin 2a, and the third as Severin 2b at skeletal maturity. Fifteen patients were found to have apparent leg shortening in the range of 3-10 mm.

## Discussion

While a concentric stable reduction is always the primary basis for a successful long-term result in the operative treatment of developmental hip dysplasia, the addition of chondroplasty to open reduction may allow for improved clinical outcomes. In this study, the expansion of the chondrolabral tissue of the acetabulum may have allowed for a more congruous developing “container” that was conducive to a progressive adaptation of the femoral head to the cartilaginous acetabular construct. According to the long-term results of this study, prompt correction of the AI with open reduction and chondroplasty is maintained to skeletal maturity without a substantial increase in the risk of AVN. Early postoperative AI can predict the late Severin grade; therefore, a correction in AI through open reduction and chondroplasty can also reduce the risk of later complications. This suggests that prompt correction of the AI may help to improve the development of all parts of the hip joint and eventually lead to near normal function.

Chondroplasty of the acetabular roof as an adjunct to open reduction for DDH has not been described in the literature; and while data on chondroplasty effects on total hip development in the setting of DDH does not exist, this study has demonstrated that chondral expansion of the osteocartilaginous edge of the acetabulum helps in the development of a more stable anatomic acetabular construct with continued improvement of the AI from the date of surgical correction, 16.97, to an average AI of 13.50 as a child grows to scale of maturity. Additionally, at the last follow-up, radiological assessments of hips by Severin’s classification were also remarkable with nearly 75% normal hips, which suggests chondroplasty may have a positive developmental effect and therefore should be indicated in the surgical treatment process for DDH in the age group from six to 18 months.

**Figure 4:**
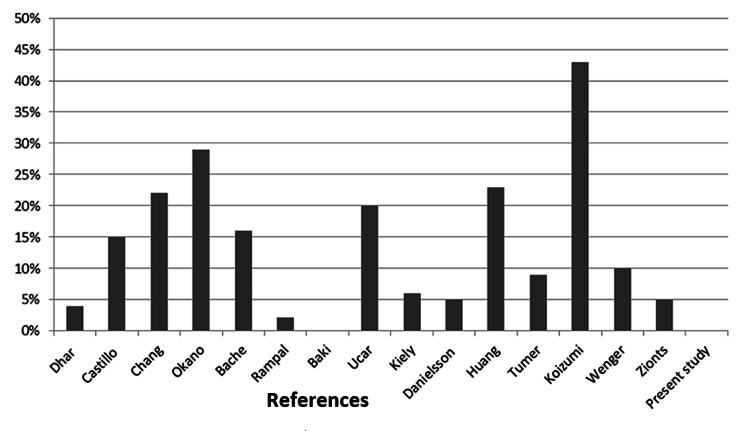
Prevalence of avascular necrosis during 15 years follow-up in the present study and other studies performed on the patients of the same age

**Figure 5:**
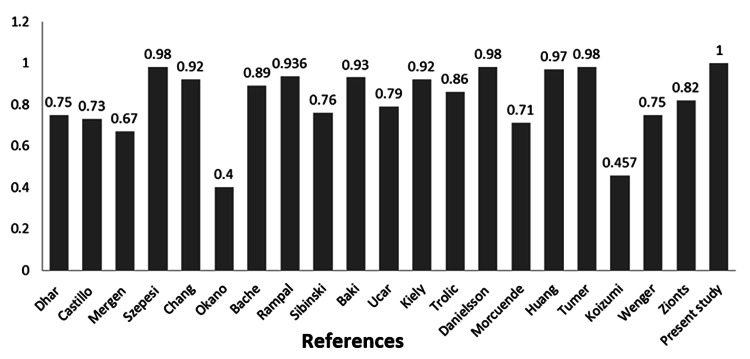
Prevalence of excellent and good radiologic results (Severin class 1 and 2) at the latest follow-up assessment in the present study and other studies performed on the patients of the same age

**Figure 6:**
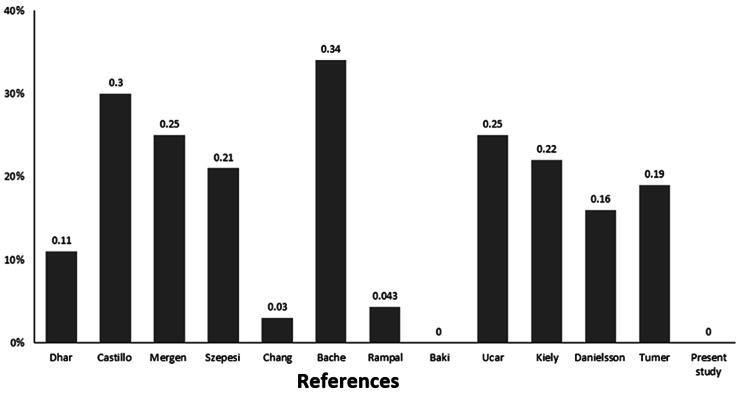
Prevalence of the need for secondary surgery during 15 years follow-up in the present study and other studies performed on the patients of the same age

Complications of the present technique in comparison with other studies in this age group are negligible. Several outcomes were compared to other studies including a radiologic assessment based on Severin's classification at the last follow-up visit, rate of secondary surgery, and occurrence of AVN with Kalamchi classification. The comparative results are summarized in Table 2 and illustrated in Figures 4, 5, 6. Dimitriou et al. [11], Dickson [12], Ghormley [13], Wade et al. [14], and Carsi et al. [15] have described similar incomplete periacetabular osteotomies in the surgical treatment of hip dysplasia. Despite subtle differences, all studies proposed adjunctive interventions that were osteotomy-based and disregarded reconstruction of acetabular chondral tissue. Most of the studies intended to produce reduction by means of stabilizing the femoral head mechanically [11-14] and only one by stimulating acetabular growth [15]. Furthermore, traditional chondroplasty is a technique that involves the debridement of intraarticular soft tissues, including cartilage and labral defects [16]. This technique has been widely cited in the knee and hip literature, particularly in studies investigating the sequelae of osteoarthritis, femoroacetabular impingement, and Legg-Calvè-Perthes disease [17-21]. In all cases, chondroplasty was performed arthroscopically with a mechanical shaver or radiofrequency energy to reduce joint friction caused by chondral labral tissue defects. Subsequent to this low safety concern procedure, patients often showed short-term improvements with low complication and reoperation rates. To our knowledge, chondroplasty has not been utilized in a manner that involves acetabular reconstruction, particularly in patients with hip dysplasia among the age group six to 18 months.

**Table 2:** Comparison of the present results with those of other studies on the basis of operations performed on the patients of the same age AVN, Avascular necrosis.

Reference	Number of hips	Age	Treatment procedure	Severin		AVN (Kalamchi classification)		Secondary surgery
				1,2	3-6	1	2-4	
Dhar et al. 1990 [5]	99	62 of 99 hips under 24 months	O.R.	75%	10%	16%	4%	11%
Castillo et al. 1990 [22]	26	4 to 15 months	Medial adductor O.R.	73%	15%	-	15%	30%
Mergen et al. 1991 [23]	31	3 to 33 months (mean = 12.1)	Medial approach O.R. + Ferguson procedure	67%	9.7%	-	-	25%
Szepesi et al. 1995 [24]	30	6 to 24 months	Ant approach O.R.	98%	0	-	-	21%
Chang et al. 2011 [4]	63	1 to 3 years	O.R. + Salter osteotomy	92%	8%	25%	22%	3%
Okano et al. 2009 [25]	45	6 to 31 months (mean = 14)	Medial approach O.R.	40%	60%	-	29%	
Bache et al. 2008 [26]	109	-	Medial approach O.R. + ligamentum teres tenodesis	89%	-	25%	16%	34%
Rampal et al. 2008 [27]	47	1 to 4.9 years	C.R.	93.6%	6.4%	-	2.1%	4.3%
Sibiñski et al. 2006 [28]	155	Mean = 14.9 months	C.R.	76%		-	-	-
Baki et al. 2005 [29]	15	13 to 30 months (mean = 20)	Medial approach O.R. + innominate osteotomy	93%	7%	0	0	0
Ucar et al. 2004 [30]	44	2 to 19 months (mean = 10.7)	Medial approach O.R.	79%	21%	-	20%	25%
Kiely et al. 2004 [31]	49	6 to 23 months (mean = 12.3)	Ferguson medial approach O.R.	92%	8%	8%	6%	22%
Trolić et al. 2002 [32]	22	7 to 29 months (mean = 15)	Medial approach O.R.	86%	14%	-	-	-
Danielsson 2000 [33]	75	2 to 64 months (mean = 10)	C.R.	98%	2%	-	5%	16%
Morcuende et al. 1997 [34]	93	2 to 50 months (mean = 14)	Anteromedial approach O.R.	71%	29%	57% without AVN	-	-
Huang et al. 1997 [35]	17	13 to 17 months	C.R.	5%	95%	-	23%	-
	32		O.R.	97%	3%	-	6%	-
Tumer et al. 1997 [36]	56	2 to 25 months (mean = 11.2)	Medial approach O.R.	98%	2%	-	8.9%	19%
Koizumi et al. 1996 [37]	35	5 to 29 months (mean = 14)	Ludloff's medial approach O.R.	45.7%	54.3%		42.9%	-
Wenger et al. 1995 [38]	20	5 to 23 months	Derotational femoral shortening osteotomy + O.R.	75%	25%	-	10%	-
Zionts et al. 1986 [39]	51	1 to 3 years	C.R. (75%)/O.R. (25%)	82%	18%	-	5%	-
Present study	35	8 to 16 months (mean = 11.9)	Chondroplasty	87.5%	0	5%	0	0

In this study, we employed a modified chondroplasty technique that may be less morbid than both osteotomy and traditional chondroplasty as this modified technique solely involves separation of the chondrolabral tissue from acetabular bone to create a construct that reinforces concentric reduction of the hip joint. Unlike traditional periacetabular osteotomies, the chondroplasty immediately stabilizes the acetabular roof and has the potential to remodel with regard to the femoral head once weight-bearing ambulation commences. The mechanical loading from the femoral head may stimulate the secondary ossification center just above the chondrolabral tissue and facilitate remodeling of the acetabular roof. There is a risk that the separation of chondrolabral tissue from bone may cause acetabular roof perforation and damage to the secondary ossification center. However, patients were followed up to skeletal maturity, and no impairments to the growing acetabulum such as leg length discrepancy (LLD) greater than 1 cm, reduced hip range of motion, or limping were recorded. In terms of LLD, it has been accepted that Salter’s osteotomy may lead to overgrowth of the treated side. Macnicol et al. reported LLD in 54 out of 148 patients who had undergone a Salter’s innominate osteotomy, which was more than 2 cm in some cases [40]. In another study, significant LLD was also reported in 2.7% of patients with DDH who were treated by simultaneous open reduction and Salter’s osteotomy [41]. LLD as a result of our technique was not greater than 1 cm in any patient. 

The most important predictor of treatment strategy and outcome in DDH is the age of presentation [6,42]; however, there are some controversies in a specific age period, such as between six and 18 months. In this age group, both closed and open reduction are possible [1,27,43,44]. Attention to the wide variety of treatment options in this specific age group reveals uncertainty as to which is optimal. This is because of the developing nature of the femoral head and the acetabulum. The femoral head becomes ossified in this age period [45]; therefore, extra pressure secondary to close reduction or interruption of blood supply following open reduction may threaten its developmental process and lead to ischemic necrosis [24]. On the other hand, if the femoral head reduction is postponed until full development of the ossific nucleus, it may lose its plasticity for remodeling to a normal configuration [45]. The second factor in maintaining the femoral head is the acetabular coverage. Although it can be improved in this age period by Salter’s osteotomy [4], Salter himself recommended to delay this procedure until 18 months of age because of the soft and small acetabular structure [46]. By employing our version of chondroplasty, an artificial space was created above the anterolateral acetabular cartilage and maintained by three wedges of bone. Therefore, not only the acetabular coverage was improved immediately, but also the secondary ossification center was stimulated to reshape the acetabulum toward a normal configuration.

While there are no specific prerequisites for the indication of chondroplasty, the step that involves separation of the chondral labral tissue from the bony edge of the acetabulum is critical, with some limitations in regard to how far the tissue can be "stretched" to create the artificial space, to which the wedge bone graft can be added. Damage to the lateral ring apophysis and tearing of the chondral labral tissue while expanding it may possibly lead to bony or cartilaginous defects at the edge of the acetabulum. These intraoperative complications may lead to a compromised final result. However, the chondral labral tissue has been shown to have pluripotent growth remodeling ability, which is expected to heal rapidly and aid in the development of a satisfactory bony contour of the anterior and lateral acetabular construct. Nevertheless, being unable to appropriately or carefully stretch the osteochondral edge of the anterior and lateral acetabular rim would be the only specific contraindication for chondroplasty supplementation. It is presumed that this skill will progress with experience to obviate intraoperative challenges and complications. In this study, separating the labral tissue from bone was accomplished successfully in all cases.

One of the main limitations of the current study was the lack of a control group. Acetabular dysplasia was the main outcome of interest in the present study and was evaluated using osseous AI. However, there is some evidence regarding the importance of the cartilaginous component of the acetabular roof in stabilizing the reduced femoral head and predicting the true potential for acetabular growth [47]. Another limitation of the present study was the lack of baseline data on the presence or absence of an ossific nucleus prior to surgery. The absence of an ossific nucleus in this age group can influence the development of post-reduction AVN. No conclusions can, therefore, be made on the influence of chondroplasty on the risk of developing AVN in patients with or without an ossific nucleus. Nonetheless, this is a preliminary report of chondroplasty for DDH as an option in the six- to 18-month age groups with a long follow-up period.

## Conclusions

Chondroplasty in conjunction with open reduction can yield a concentric reduction with improved acetabular coverage that facilitates acetabular remodeling that is sustained until skeletal maturity. Prompt correction through this procedure may help to improve the development of the hip and lead to near normal function as demonstrated by improved mean AI and Severin scores at the last follow-up. With low complication and reoperation rates, this procedure could be considered as a surgical treatment option for DDH in patients between the age of six and 18 months.
